# Nursing Talent Management Models: A Scoping Review

**DOI:** 10.3390/nursrep16100346

**Published:** 2026-09-23

**Authors:** Telma Quaresma, Maria Céu Marques, Liliana Mota

**Affiliations:** 1 CHRC—Comprehensive Health Research Centre, University of Évora, 7004-516 Évora, Portugal; telma.quaresma@ulsalg.min-saude.pt (T.Q.); mcmarques@uevora.pt (M.C.M.); 2 Algarve Local Health Unit—Hospital Unit of Portimão, Department of Education, Innovation and Research, 8500-338 Portimão, Portugal; 3 São João de Deus School of Nursing, University of Évora, 7004-516 Évora, Portugal; 4 Portuguese Red Cross Northern Health School, 3720-126 Oliveira de Azeméis, Portugal; 5 RISE-Health, Faculty of Medicine, University of Porto, 4200-319 Porto, Portugal

**Keywords:** talent management, nursing, hospital, scoping review

## Abstract

**Background:** Healthcare systems worldwide are facing an unprecedented nursing workforce crisis driven by staff shortages, population ageing, increasing care complexity, and difficulties in retaining qualified professionals. Although talent management has emerged as a strategic organisational approach to address these challenges, its application within hospital nursing remains fragmented and has not yet been systematically synthesised. **Objective:** To map the available scientific evidence on organisational approaches to talent management in hospital nursing. **Methods:** A scoping review was conducted following the Joanna Briggs Institute methodology and reported according to the PRISMA-ScR guidelines. A comprehensive search was performed on 20 February 2026 across MEDLINE, CINAHL^®^ Plus with Full Text, Nursing & Allied Health Collection, MedicLatina, CENTRAL, CDSR, LILACS, SciELO, RCAAP, and additional grey literature sources. Eligibility criteria were established using the Population–Concept–Context (PCC) framework. Study selection and data extraction were independently conducted by two reviewers. **Results:** From 8534 records identified, 11 eligible records representing 10 independent studies were included. Four core Talent Management processes were identified: talent identification, development, retention and evaluation. The interpretative synthesis further distinguished organisational antecedents, including participative leadership, development-oriented cultures, professional recognition and academic–practice partnerships; relational and motivational mechanisms, including work engagement, psychological contract fulfilment and social capital; and workforce and organisational outcomes, particularly retention intention, leadership development and organisational commitment. These findings informed the development of an integrated conceptual framework for Talent Management in hospital nursing. **Conclusions:** The available evidence suggests that Talent Management may be more appropriately conceptualised as an integrated organisational capability rather than as a set of isolated human resource practices. This review provides an integrated mapping of the available evidence on Talent Management models specifically focused on hospital nursing, offering a conceptual foundation to support future research, organisational strategies and model development.

## 1. Introduction

Health systems worldwide are currently facing an unprecedented crisis in nursing human resources. According to the World Health Organization, the global shortage of nurses is estimated at 5.9 million professionals, disproportionately affecting low- and middle-income countries and compromising the sustainability of health systems [1]. At the same time, the ageing of the workforce, with around a quarter of nurses in OECD countries approaching retirement age, together with increasing care complexity and the growing difficulty in recruiting and retaining qualified professionals, further aggravates this scenario [2,3]. These challenges demonstrate that the sustainability of health organisations depends not only on recruitment capacity but also on the ability to identify, develop and retain highly qualified professionals. Human capital constitutes the most valuable resource of health organisations, with talent being one of the main differentiating factors of organisational performance [4,5]. However, the growing shortage of highly qualified professionals and the difficulty in ensuring their retention render the adoption of traditional human resource management practices insufficient [6,7,8]. In this context, Talent Management emerges as a strategic approach, defined as a continuous and integrated process of attracting, identifying, developing, retaining and valuing professionals whose contribution is decisive for organisational success [9,10]. Unlike conventional human resource management approaches, Talent Management seeks to align professionals’ potential with the organisation’s strategic objectives, integrating practices such as strategic recruitment, competency development, mentoring, performance recognition and succession planning [11,12]. Despite growing academic interest, the conceptualisation of talent and Talent Management remains controversial. Some authors define talent based on the three C’s theory: competence, commitment and contribution [13], while others characterise it by the capacity to deliver superior performance and solve complex problems in highly demanding contexts [14]. Similarly, Talent Management is understood either as a strategy for generating sustainable competitive advantage or as the process of placing the right person in the right role to achieve the organisation’s strategic objectives [15]. This conceptual diversity has been identified as one of the main obstacles to the consistent implementation of Talent Management in organisations, hindering the definition of coherent, comparable and measurable policies [16].

The international literature nevertheless identifies two major conceptual streams underpinning Talent Management [17,18,19]. The Exclusive Talent Management perspective, proposed by Collings and Mellahi [17], argues that organisational resources should be prioritised for a restricted group of professionals considered high-potential or holding strategic positions, capable of generating sustainable competitive advantage. In contrast, the Inclusive Talent Management perspective holds that all employees possess the potential to develop talent, provided they are given adequate opportunities for learning, development and professional progression [18,19]. Although these approaches have been widely discussed in the management literature [17,18,19], it remains unclear how they have been operationalised in hospital nursing and which organisational models reflect each of these perspectives, justifying the need for a critical synthesis of the available evidence.

In nursing, Talent Management assumes particular strategic relevance, given the centrality of nurses in complex care processes and their disproportionate exposure to burnout, moral distress and professional attrition. The available evidence suggests that Talent Management practices applied to nursing teams are associated with improvements in professional recognition, clinical leadership, work motivation and patient safety outcomes [20,21]. Additionally, Talent Management has been associated with an improved organisational climate, reduced turnover intention and burnout, and strengthened organisational commitment among nurses [22,23]. In the hospital context, where operational demands and workforce complexity are higher, structured talent management models constitute a promising framework for aligning individual professional development with organisational performance objectives [8,24]. Despite these promising results, it remains unclear which organisational components characterise the different Talent Management models implemented in hospital nursing, as well as how these have been operationalised and evaluated across different organisational contexts.

A talent management model can be conceptualised as an integrated organisational configuration that articulates policies, practices and processes aimed at identifying, developing, retaining and evaluating professionals considered strategic to organisational performance [25,26,27]. These models are distinguished from isolated human resource management interventions by their systemic and strategic nature, integrating dimensions such as participative leadership, a development-oriented organisational culture, succession planning, continuous professional development and mechanisms for performance and retention evaluation [28,29]. Despite the growing volume of research on Talent Management in healthcare, the available evidence remains scattered across different conceptual frameworks, organisational models and implementation contexts. Existing reviews tend to address Talent Management transversally across various health professions or human resource management systems, which does not allow an integrated understanding of which models are specifically applied to hospital nursing, what their structuring components are, which organisational outcomes are consistently associated with their implementation, and what the main knowledge gaps are. This absence of synthesis hinders the comparison between approaches, limits the development of evidence-based organisational models, and constrains the formulation of people management policies tailored to the specific needs of hospital nursing. Given the conceptual and methodological heterogeneity identified in the literature, a Scoping Review constitutes the most appropriate methodological approach for systematically mapping the available evidence, identifying knowledge gaps and supporting the development of future organisational Talent Management strategies in nursing. This Scoping Review therefore aimed to map the evidence on organisational approaches to Talent Management in hospital nursing, including conceptual models, implementation strategies, organisational practices, reported outcomes and knowledge gaps.

## 2. Materials and Methods

A Scoping Review was conducted to systematically map the available scientific evidence on organisational approaches to Talent Management in hospital nursing, to identify knowledge gaps and to guide future lines of research. This type of review is particularly suited to situations where the available evidence is conceptually heterogeneous, includes different methodological designs, and aims to explore the extent, nature and characteristics of the existing literature, rather than answering a question about the effectiveness of an intervention, which is the typical objective of systematic reviews [30]. Given the diversity of conceptual models, organisational strategies and implementation contexts identified in the literature on Talent Management in nursing, conducting a Scoping Review proved to be the most appropriate methodological approach for addressing the objective of this study [30,31].

The methodology adopted followed the recommendations of the Joanna Briggs Institute (JBI) for Scoping Reviews [30], and reporting was guided by the PRISMA-ScR checklist (Preferred Reporting Items for Systematic Reviews and Meta-Analyses extension for Scoping Reviews, Table S1) [31]. The protocol for this review was previously registered on the Open Science Framework (OSF), ensuring transparency, traceability and methodological reproducibility (10.17605/OSF.IO/GW2KR). No amendments were made to the review question, eligibility criteria, or analytical approach following protocol registration. Search strategies were subsequently reviewed and technically refined to ensure database-specific syntax and consistency across information sources.

### 2.1. Eligibility Criteria

Eligibility criteria were defined a priori based on the PCC strategy (Population, Concept and Context), ensuring alignment between the research question, the search strategy and the study selection process, as recommended by the Joanna Briggs Institute [30,31]. The use of this framework made it possible to clearly delimit the scope of the review, ensuring consistency between the population of interest, the concept under study and the organisational context analysed. The eligibility criteria were established prior to the literature search and applied consistently throughout the study selection process. The research question guiding the review was as follows: “What is the available scientific evidence on organisational approaches to Talent Management directed at nurses in the hospital setting?” Studies published in English, Portuguese or Spanish were eligible for inclusion; records published in other languages were excluded during the screening process. The inclusion and exclusion criteria, organised according to the PCC framework, are presented in Table 1.

### 2.2. Information Sources and Search Strategy

The literature search was conducted on 20 February 2026 across international electronic databases specialising in nursing, health sciences and evidence-based medicine: CINAHL^®^ Plus with Full Text (via EBSCO), MEDLINE (via PubMed), Nursing & Allied Health Collection (via EBSCO), MedicLatina (via EBSCO), Cochrane Central Register of Controlled Trials (CENTRAL) and the Cochrane Database of Systematic Reviews (CDSR). These databases were selected as the main international sources of scientific literature in the fields of nursing, health sciences, management and clinical evidence, allowing comprehensive coverage of the topic under study.

Additionally, a grey literature search was conducted on 20 February 2026 using DANS Easy, RCAAP (Portuguese Open Access Scientific Repository), SciELO and LILACS (via BVS) platforms. The grey literature search was included with the aim of reducing publication bias and identifying potentially relevant evidence not indexed in traditional bibliographic databases, as recommended for scoping reviews [30]. A manual search of the reference lists (backward reference tracking) of the included studies was also carried out, in order to identify potentially relevant studies not retrieved by the electronic search strategy. The combination of these information sources sought to maximise the sensitivity of the search strategy, ensuring comprehensive coverage of the published literature and of the grey literature relevant to answering the research question. The search strategy was developed based on controlled vocabulary, including MeSH descriptors where applicable, and free-text keywords combined with Boolean operators (AND, OR), and was adapted to the syntax and indexing characteristics of each database. Following revision, the search strategies were independently reviewed by a second reviewer with expertise in bibliographic search methodology, with particular attention to Boolean structure, field syntax, controlled vocabulary, database-specific adaptations and consistency across information sources. Following this independent review, the corrected search strategies reported in Table 2 were executed across all information sources on 20 February 2026. Study selection was performed by two independent reviewers, with discrepancies resolved by consensus.

### 2.3. Study Selection

Records obtained from the databases were exported to the Zotero reference manager (version 6.0.36), where duplicates were systematically identified and removed. Non-duplicate records were subsequently imported into the Rayyan platform (2023 version) to support the screening process. During the initial screening phase, the reviewers worked independently using Rayyan’s blind mode function, reducing the risk of influence between eligibility decisions.

Study selection took place in two sequential and independent stages: (i) screening of titles and abstracts, based on the previously defined eligibility criteria; (ii) reading and analysis of the full texts of potentially eligible studies. Both stages were conducted independently by two reviewers. Discrepancies were resolved by consensus between the two reviewers, and it was not necessary to involve a third reviewer, as all disagreements were resolved by consensus.

The reasons for exclusion during the full-text analysis stage were systematically documented.

A total of 36 reports were sought for retrieval, of which two could not be retrieved. Consequently, 34 full-text reports were assessed for eligibility. Of these, 26 were excluded: 10 did not meet the Population criterion (non-nursing samples or nursing students as the primary population), 9 did not meet the Concept criterion (focus on clinical or technical competencies without an explicit Talent Management framework), and 7 did not meet the Context criterion (non-hospital settings). These figures correspond to the exclusion counts reported in the PRISMA-ScR flow diagram (Appendix A). The completed PRISMA-ScR checklist is available as Supplementary Material.

For reporting purposes, each eligible publication was retained as an included record in the review. Where multiple publications were found to originate from the same underlying study, these were identified as connected reports and were not interpreted as independent sources of evidence.

### 2.4. Data Extraction

Data extraction was carried out independently by two reviewers. The extraction instrument was aligned with the review’s objectives and with the PCC strategy, in accordance with the recommendations of the Joanna Briggs Institute [30]. The extraction instrument was iteratively refined throughout the process whenever a need for clarification was identified, without altering the review’s objectives. The complete extraction instrument is available as Supplementary Material.

Discrepancies between reviewers during the extraction phase were resolved by consensus. Information was collected on title, authors, year of publication, country, study type, main objective, main results, sample/participants, setting, recruitment, measures/data collection, analysis, and detailed numerical or qualitative findings (Appendix B). In accordance with JBI recommendations for scoping reviews, no critical appraisal of the methodological quality of the included studies was performed [30]. This procedure aimed to ensure the consistency, transparency and reproducibility of the data extraction process.

### 2.5. Data Analysis and Presentation

The extracted data were analysed descriptively and synthesised using a hybrid deductive–inductive thematic categorisation approach. The four core Talent Management processes—identification, development, retention and evaluation—were used as a priori organising categories derived from pre-existing Talent Management models described in the literature. Additional organisational antecedents, relational and motivational mechanisms, outcomes and maturity patterns were subsequently generated inductively through iterative reading, comparison and synthesis of the included studies. The categories were progressively refined by two reviewers in joint analysis sessions until consensus was reached. Disagreements in categorisation were discussed and resolved by consensus between the reviewers.

Building on this thematic categorisation, a second phase of interpretative synthesis was undertaken, in which the two reviewers jointly examined the identified themes, organisational antecedents, mechanisms and outcomes across the included studies in order to identify recurring patterns in the degree of integration, coordination and strategic alignment of Talent Management practices. This interpretative process, conducted iteratively until consensus was reached, informed the development of the integrated conceptual framework (Section 3.6) and the proposed Nursing Talent Management Maturity Model (Section 4.6). A mapping table linking each included study to the corresponding components of the conceptual framework and, where applicable, to the maturity level to which its findings were associated, is provided as Appendix C to enhance the transparency and traceability of this interpretative process.

Coding was governed by explicit a priori rules. A finding was coded to one of the four core Talent Management processes when it directly described an activity aimed at recognising potential (identification), building competencies (development), sustaining workforce commitment or intention to stay (retention), or measuring or appraising talent-related constructs (evaluation). Findings describing a contextual condition enabling Talent Management, rather than a management process itself, were coded as organisational antecedents; findings describing an explanatory pathway between a practice and an outcome were coded as relational/motivational mechanisms; and findings describing a distal consequence were coded as workforce or organisational outcomes. For the proposed maturity model, each study was assigned to the level reflecting the highest degree of Talent Management integration explicitly evidenced in its findings. Level 1 (Administrative Human Resource Management) was assigned when Talent Management was absent as a structured organisational approach and workforce management was predominantly administrative or reactive. Level 2 (Talent Practices) was assigned when at least one explicit Talent Management practice was identified, but practices remained isolated, fragmented or limited to specific individuals, units or programmes. Level 3 (Integrated Talent Management) was assigned when multiple Talent Management processes were coordinated within a shared organisational structure or programme. Level 4 (Strategic Talent Capability) was assigned when Talent Management was explicitly integrated with organisational strategy, workforce planning and systematic evaluation. Level 5 (Learning Health Organisation) was defined as the presence of Level 4 characteristics together with continuous organisational learning, data-informed feedback and systematic adaptation of Talent Management practices. Two reviewers applied these criteria independently to each included study, and disagreements were resolved by consensus, as detailed for each study in the Rationale column of Appendix C. It should be noted that the four core processes empirically coded across the included studies—identification, development, retention and evaluation—remain the only a priori categories supported by the coded evidence (Appendix C); no included study was coded as addressing a distinct ‘deployment’ process. ‘Deployment’ is introduced later, in Section 3.6 as an inductively derived conceptual refinement of the ‘development’ process, denoting the organisational assignment of identified talent to differentiated roles once developed, and again in Section 4.9 as a related practice recommendation. This addition was not derived from a distinct set of empirical codes and should therefore be interpreted as a theoretical refinement of the framework rather than as a fifth a priori coding category, with the same caution applied to the other inductively derived framework components described above. Accordingly, deployment was not used as an independent coding category or as a separate criterion for assigning studies to maturity levels.

To enhance analytical transparency and traceability, Appendix D documents, for each included study, an illustrative extracted finding, its assigned code, category and corresponding higher-order construct.

The synthesis of results was conducted narratively, as recommended by the JBI for scoping reviews in which the heterogeneity of the included studies does not allow for meta-analysis [30]. Disagreements in categorisation were discussed and resolved by consensus between the reviewers.

Throughout the synthesis, the methodological nature and evidential role of each included source were considered explicitly. Empirical findings were distinguished from conceptual, methodological, secondary and practice-based contributions, and the latter were used primarily to inform interpretation, contextualisation and framework development rather than being treated as equivalent empirical evidence. The term “mechanism” is used in this review to denote an evidence-informed hypothesised pathway linking Talent Management practices to reported outcomes and does not imply an established causal mechanism unless causality was directly tested in the original study.

The knowledge gaps identified throughout the synthesis process were systematically documented, constituting a central contribution of this scoping review towards guiding future research.

The data are presented in the form of a PRISMA-ScR flow diagram (Appendix A).

### 2.6. Ethical Considerations

As this is a literature review based on secondary data, approval by an ethics committee was not required.

## 3. Results

This scoping review included 11 eligible records addressing Talent Management in hospital nursing (corresponding to 10 independent studies, as references [32,33] report the same underlying research programme and sample). The records reveal still-emerging evidence, comprising different methodological designs, including quantitative studies (*n* = 3), qualitative studies (*n* = 2), methodological studies (*n* = 2), one conceptual analysis, one systematic review, one review and one practice article, showing that this domain combines both conceptual development and empirical research. Given the methodological heterogeneity of the records included, their findings were interpreted according to their evidential role rather than treated as equivalent forms of evidence. Empirical studies were used primarily to identify reported associations, experiences and workforce outcomes; methodological studies informed the measurement and evaluation dimension; conceptual and practice-based sources contributed to the identification and interpretation of organisational components and implementation approaches; review evidence was used to contextualise broader patterns across the literature. Accordingly, empirically reported findings were distinguished from the conceptual relationships generated through the interpretative synthesis.

The 11 eligible records originated from seven countries. The United States, the United Kingdom, Egypt and Türkiye accounted for the largest number of publications (*n* = 2; 18.2% each), while Canada, Malawi and Cyprus each contributed one record (*n* = 1; 9.1%). This geographical distribution shows that Talent Management in nursing has attracted interest across different international contexts, spanning countries in North America, Europe, Africa and the Middle East. However, the limited concentration of studies per country suggests that this scientific domain remains at a developmental stage, with no consolidated body of research yet established in specific contexts or health systems.

The chronological distribution of the eligible records indicates an increasing concentration of publications in recent years. Records published between 2005 and 2014 accounted for three publications (*n* = 3; 27.3%), those published between 2015 and 2020 for two (*n* = 2; 18.2%), and those published between 2021 and 2025 for six (*n* = 6; 54.5%). Earlier publications focused mainly on conceptual, organisational and practice-oriented approaches to Talent Management, whereas more recent records increasingly included empirical studies, systematic synthesis and the development and validation of measurement instruments. This pattern suggests a gradual expansion of the field from predominantly conceptual and practice-based contributions towards greater empirical and methodological development.

The older models are predominantly strategic and conceptual in nature, seeking to justify the importance of human capital as a source of competitive advantage. In contrast, the more recent models present a more operational orientation, integrating recruitment, development, performance evaluation, succession and retention processes within a continuous organisational approach.

### 3.1. Interpretative Structure of Nursing Talent Management

The descriptive mapping identified four core processes within the Talent Management approaches reported in the included studies: talent identification, talent development, talent retention and talent evaluation. These processes were not represented with equal frequency or depth. Talent retention was the most frequently identified component, being addressed in seven independent studies, followed by talent development in five, talent identification in two, and talent evaluation in one independent study reported across two connected records [32,33].

Beyond this descriptive categorisation, the interpretative synthesis revealed that these processes were embedded within a broader organisational architecture. Three analytically distinct but interrelated levels were identified: organisational antecedents and enabling conditions, core Talent Management processes, and relational and motivational mechanisms through which the reported outcomes may emerge.

Organisational antecedents included participative leadership, a development-oriented organisational culture, professional recognition, career development structures, academic–practice partnerships and opportunities for professional participation. These conditions appeared to create the organisational environment required for Talent Management practices to be implemented consistently.

Relational and motivational mechanisms included work engagement, psychological contract fulfilment, professional confidence, organisational commitment, interpersonal relationships and social capital. The reported associations were consistent with possible indirect pathways linking Talent Management practices to workforce outcomes through these mechanisms, rather than with a simple direct relationship.

The principal reported outcomes included retention intention, leadership development, professional engagement and organisational commitment. Evidence concerning actual retention, innovative behaviour, organisational performance and patient-related outcomes was more limited or inconsistent. These findings informed the development of an integrated conceptual framework for Talent Management in hospital nursing (Figure 1).

#### 3.1.1. Talent Identification

Talent identification was described as a systematic process of recognising professionals with high performance potential, distinctive competencies or leadership capacity. It was predominantly addressed in conceptual studies and organisational frameworks and positioned as an early stage of Talent Management intended to align individual potential with organisational strategic objectives.

Nowak and Scanlan [34] identify the early identification of emerging leaders as a central attribute of Talent Management in nursing. Complementarily, Nowak and Scanlan [34] integrate talent identification as the first element of a systemic Talent Management model, while Gül [32] incorporates this dimension into the conceptual model that underpinned the development of the Nurses’ Perception of Talent Management Scale (NPTMS).

#### 3.1.2. Talent Development

Talent development constitutes the most consistent dimension in the literature reviewed. The strategies described include structured training programmes, mentoring, competency development, career planning, clinical leadership and continuous learning, being common to most conceptual and organisational models. In the hospital context, Haines [35] identifies leadership development, performance evaluation, the definition of career pathways and professional recognition as critical factors. Similarly, Cadmus et al. [36] reported that academic–hospital partnership programmes were associated with improved professional transition, confidence and clinical competence among newly graduated nurses, as well as early retention.

#### 3.1.3. Talent Retention

Talent retention was predominantly associated with empirical studies and organisational outcomes. Talent Management practices were consistently associated with organisational commitment, work engagement, psychological contract fulfilment and nurses’ intention to remain within organisations. Ellethiey et al. [37] reported an association between perceptions of Talent Management practices and psychological contract fulfilment, with cordial interpersonal relations statistically mediating this relationship. Fisher et al. [38] reported that nurses participating in a structured Talent Management initiative perceived the programme as supporting their retention intentions, while networking and social capital emerged as relevant relational factors. Similar results were observed by Elhaddad et al. [39], who associated Talent Management with higher levels of work engagement and retention intention. The Magnet model described by Upenieks [40] also emphasised participative leadership, professional autonomy, recognition and continuous development as fundamental strategies for attracting and retaining nurses.

#### 3.1.4. Talent Evaluation

Talent evaluation proved to be the least explored component. Only a small number of studies presented specific instruments or indicators designed to measure Talent Management practices, revealing an important gap in the literature regarding the systematic evaluation of these interventions. In parallel, Gül and Sönmez [33] developed and validated the Nurses’ Perception of Talent Management Scale (NPTMS), currently the only instrument specifically designed to assess nurses’ perceptions of Talent Management practices. Despite these contributions, the literature continues to highlight the absence of multidimensional instruments that are culturally adapted and validated across different organisational contexts, constituting an important gap identified in this review. The talent management components identified in the narrative analysis are summarised in Table 3.

### 3.2. Organisational Antecedents and Enabling Conditions

The narrative analysis identified a set of organisational strategies used to operationalise Talent Management in hospital nursing. Despite the diversity of organisational contexts and conceptual models used, the studies converged on the identification of five main groups of strategies: participative leadership, professional development, professional recognition and appreciation, strengthening of collaborative networks and social capital, and partnerships between health and educational institutions.

Participative leadership emerged as one of the strategies most frequently associated with talent development and retention. The included sources associated leadership styles that promote professional autonomy, participation in decision-making and recognition of individual contribution with organisational commitment and environments supportive of talent development [35,40].

Continuous professional development likewise constituted a cross-cutting component of the identified models. The strategies include structured training programmes, competency development, career planning, performance evaluation and the preparation of future leaders, enabling nurses’ professional growth to be aligned with organisations’ strategic objectives [35].

Professional recognition emerged as another relevant strategy, frequently associated with performance recognition, career progression opportunities and the strengthening of motivation and organisational commitment [35,40].

The narrative analysis further highlighted the importance of organisational networking and social capital as facilitating mechanisms for talent retention. In particular, Fisher et al. [38] reported that participation in professional networks and the strengthening of interpersonal relationships were associated with organisational integration and retention intention.

Finally, the partnerships between academic institutions and health organisations were identified as strategies facilitating the attraction, integration and development of new professionals. Cadmus et al. [36] reported that academic–hospital partnership programmes were associated with professional transition, clinical competence, professional confidence and early nurse retention.

Together, these findings suggest that the operationalisation of Talent Management in hospital nursing involves an integrated approach combining leadership, professional development, recognition, organisational collaboration and career planning.

### 3.3. Relational and Motivational Mechanisms

The interpretative synthesis identified several relational and motivational mechanisms that may help explain how Talent Management practices are associated with workforce outcomes. These mechanisms included work engagement, psychological contract fulfilment, professional confidence, interpersonal relationships, social capital and organisational commitment.

Ellethiey et al. [37] showed that the relationship between Talent Management practices and psychological contract fulfilment was influenced by the quality of interpersonal relationships and by generational differences. Fisher et al. [38] identified professional networks and social capital as mechanisms supporting nurses’ organisational integration and retention intentions. Elhaddad et al. [39] reported associations between positive perceptions of Talent Management, work engagement and intention to remain within the organisation. Academic–practice partnerships were also associated with increased professional confidence and competence among newly qualified nurses [36].

Taken together, these findings suggest that Talent Management practices may not be directly associated with retention and other workforce outcomes. Rather, the reported associations may be consistent with pathways involving the strengthening of nurses’ professional engagement, relational integration, confidence, perceived organisational support and commitment. However, these relationships were not consistently tested through longitudinal or causal designs and should therefore be interpreted as evidence-informed conceptual pathways.

### 3.4. Measurement Instruments

The evaluation of Talent Management in nursing remains an underdeveloped area, with a scarcity of instruments specifically designed to measure this construct.

Among the included studies, only one instrument was developed and validated specifically for this context. Gül and Sönmez [33] developed the Nurses’ Perception of Talent Management Scale (NPTMS), comprising 26 items within a unidimensional structure, showing adequate psychometric properties for assessing nurses’ perceptions of organisational Talent Management practices.

Despite these contributions, the narrative analysis demonstrated a limited availability of multidimensional instruments, culturally adapted and validated across different health contexts, thereby limiting the comparability of results between organisations and countries.

### 3.5. Workforce and Organisational Outcomes

Five main categories of outcomes reported in the included studies were identified: nurse retention, work engagement, leadership development, strengthening of the psychological contract, and innovative behaviour. The main organisational outcomes associated with talent management identified in the literature are summarised in Table 4.

The included studies consistently reported associations between structured Talent Management practices and greater nurse retention intention within organisations [36,37,38,39,40]. In parallel, positive associations were found with higher levels of work engagement, organisational commitment and strengthening of the psychological contract [37,40].

Leadership development also emerged as a relevant outcome, being considered a central element in ensuring organisational continuity and preparing future clinical leaders [34,35]. In contrast, Dzimbiri and Molefi [42] found no evidence of a significant impact of Talent Management practices on innovative behaviour, attributing this finding to the absence of structured organisational Talent Management policies in the hospitals studied.

Some constructs reported as outcomes in the primary studies, particularly work engagement and psychological contract fulfilment, were subsequently interpreted in the integrated synthesis as potential relational or motivational mechanisms linking Talent Management practices to more distal workforce and organisational outcomes.

### 3.6. Integrated Nursing Talent Management Framework

Based on the descriptive mapping and interpretative synthesis, an integrated conceptual framework was developed to represent the organisational architecture of Talent Management in hospital nursing. The framework positions Talent Management as a dynamic organisational capability operating through the interaction of organisational antecedents, core management processes, relational and motivational mechanisms, and workforce and organisational outcomes.

As illustrated in Figure 1, leadership, organisational culture, professional recognition, career structures and academic–practice partnerships provide the enabling conditions for the implementation of Talent Management processes. These processes include talent identification, development, deployment, retention management and evaluation. Their relationship with workforce outcomes appears to be supported by mechanisms such as engagement, psychological contract fulfilment, professional confidence, social capital and organisational commitment. Deployment is an inductively derived refinement of the development process rather than a fifth a priori coding category.

The framework should be interpreted as an evidence-informed conceptual synthesis rather than as a validated causal model. The relationships represented require empirical testing through longitudinal, implementation and multilevel research designs.

While the four core Talent Management processes—identification, development, retention and evaluation—were used as a priori organising categories derived from pre-existing Talent Management models, the organisational antecedents, relational and motivational mechanisms, outcomes and maturity patterns were generated inductively through the interpretative synthesis. The structural interrelationships represented in Figure 2 should therefore be regarded as evidence-informed propositions rather than as direct empirical results.

### 3.7. Knowledge Gaps Identified

Four main gaps were identified in research on Talent Management in hospital nursing.

First, there remains an absence of conceptual consensus regarding the definition of Talent Management and the components that should form part of the respective organisational models [34,40].

Second, there was a scarcity of models specifically developed and empirically tested for hospital nursing, with adaptations of models originating from business management predominating [33].

The third gap relates to the limited availability of evaluation instruments, with only one instrument validated specifically to measure nurses’ perceptions of Talent Management [33].

A predominance of cross-sectional and descriptive studies was observed, with a limited number of longitudinal studies or studies evaluating the impact of Talent Management strategies over time [35,36,38,39,40,41,42].

## 4. Discussion

The findings of this review suggest that Talent Management in hospital nursing extends beyond a collection of isolated human resource practices. The interpretative synthesis suggests that it can be conceptualised as a dynamic organisational capability emerging from the interaction between organisational antecedents, Talent Management processes, relational and motivational mechanisms, and workforce and organisational outcomes. This interpretation advances the existing literature by explaining not only which practices have been reported, but also the organisational conditions and mechanisms through which these practices may contribute to workforce sustainability. This perspective is consistent with Mitosis et al. [41], who highlighted the limited empirical development of Talent Management in healthcare and the need to move beyond fragmented initiatives towards integrated, contextualised and measurable organisational approaches.

### 4.1. From Fragmented Practices to Organisational Capability

Previous nursing literature has primarily described individual Talent Management practices, including talent identification, professional development, performance evaluation and retention. However, the findings of this review suggest that the strategic value of Talent Management does not result from the isolated implementation of any one of these practices. Rather, its value appears to depend on their integration within an organisational system aligned with nursing workforce needs and institutional objectives.

The conceptualisation of Talent Management as a dynamic organisational capability is supported by Nowak and Scanlan [34], who describe it as a cyclical and adaptive process involving the identification, development and retention of nursing talent. The present review extends this perspective by suggesting that these processes are embedded within a broader organisational architecture. Their implementation appears to depend on antecedents such as participative leadership, a development-oriented culture, professional recognition, career structures and academic–practice partnerships. Their relationships with workforce outcomes may, in turn, be explained through relational and motivational mechanisms, including work engagement, psychological contract fulfilment, professional confidence, social capital and organisational commitment.

Talent Management should therefore be distinguished from isolated professional development, recruitment or retention interventions. Although these activities may form part of Talent Management, they do not constitute a comprehensive model unless they are strategically aligned, mutually reinforcing and supported by appropriate organisational structures. This distinction is important because hospitals may implement individual Talent Management practices without developing the organisational capability required to sustain them or generate meaningful workforce outcomes.

### 4.2. Organisational Architecture Supporting Talent Management

The findings indicate that the antecedents identified across the included literature are better understood not as discrete Talent Management interventions, but as elements of an enabling organisational architecture. Their relevance lies in creating the conditions under which talent identification, development, deployment and retention can be integrated and sustained within nursing workforce management. This interpretation shifts attention from the presence of individual practices to the organisational capacity to coordinate them within a coherent system.The Magnet model described by Upenieks [40] illustrates the importance of aligning recruitment practices, participative leadership, professional autonomy, recognition and development opportunities with organisational culture. Rather than representing independent retention initiatives, these elements may be interpreted as components of an enabling organisational architecture that supports the attraction, integration and continued development of nurses.

Academic–practice partnerships provide another example of an organisational structure supporting Talent Management. Cadmus et al. [36] showed that structured transition programmes developed through collaboration between educational institutions and hospitals supported the confidence, competence and professional integration of newly qualified nurses. These partnerships may therefore function not only as educational interventions but also as organisational antecedents of early career development and retention.

Similarly, Haines [35] identified team leadership, effective performance evaluation, clear career pathways, professional participation and recognition as interconnected conditions for the development of nursing talent. These findings suggest that Talent Management requires organisational investment in both formal structures and the professional practice environment. However, the included literature provides limited information regarding how these antecedents should be governed, financed, operationalised or adapted across different hospital systems. Implementation strategies therefore remain insufficiently developed and tested.

### 4.3. Relational Pathways Linking Talent Management to Workforce Outcomes

The principal interpretative implication of these findings is that several constructs reported alongside Talent Management may be more appropriately understood as intermediate pathways rather than final organisational outcomes. This distinction helps explain why similar Talent Management practices may be associated with different retention-related outcomes across organisational contexts. Ellethiey et al. [37] identified an association between nurses’ perceptions of Talent Management practices and psychological contract fulfilment. Interpersonal relationships acted as a mediating mechanism, while generational membership influenced the strength and direction of these relationships. These findings indicate that the effectiveness of Talent Management may depend not only on the availability of development or career opportunities but also on how nurses interpret the reciprocal relationship between themselves and their organisation.

Fisher et al. [38] similarly found that nurses perceived Talent Management initiatives as supporting their intention to remain within the organisation. Professional networks and social capital appeared to facilitate organisational integration and access to career-related opportunities. This finding extends understanding of retention beyond contractual or financial incentives by showing that professional relationships, visibility and participation in organisational networks may influence nurses’ experiences of belonging and future career prospects.

Elhaddad et al. [39] also reported associations between positive perceptions of Talent Management, work engagement and retention intention. Together, these findings suggest that Talent Management may not influence retention directly. Development opportunities, professional recognition, participation in organisational decisions and supportive interpersonal relationships may first strengthen engagement, confidence, perceived organisational support and professional attachment, which subsequently contribute to nurses’ willingness to remain.

Nevertheless, most of these relationships were examined through cross-sectional, perception-based or qualitative designs. They should therefore be interpreted as evidence-informed conceptual pathways rather than as established causal mechanisms. Longitudinal and multilevel studies are required to determine whether changes in Talent Management practices produce corresponding changes in engagement, psychological contract fulfilment, organisational commitment and workforce retention.

### 4.4. Interpreting Workforce Outcomes and the Limits of Current Evidence

The evidence base is strongest for proximal, perception-based workforce outcomes and substantially weaker for objectively measured organisational and patient outcomes. This distinction is important because work engagement, psychological contract fulfilment, organisational commitment and intention to remain should not be interpreted as equivalent to actual retention, turnover reduction or organisational performance. As no formal methodological quality appraisal was undertaken, this observation reflects the frequency and consistency with which these associations were reported across included studies, rather than a judgement of their methodological strength or evidential weight; findings should accordingly be read as descriptive associations rather than as evidence of comparative evidential quality. In contrast, evidence concerning actual retention, turnover reduction, innovative behaviour, organisational performance and patient outcomes remains limited or inconsistent.

This distinction is particularly important in relation to retention. Several included studies assessed nurses’ intention to remain rather than objectively measured retention or turnover [37,38,39]. Although intention to stay is an important attitudinal indicator, it should not be treated as equivalent to actual workforce behaviour. The relationship between intention and behaviour may be influenced by employment opportunities, remuneration, career progression, family circumstances, labour market conditions and organisational restructuring.

The findings reported by Dzimbiri and Molefi [42] are particularly relevant because they challenge the assumption that Talent Management practices automatically generate higher-order organisational outcomes. The absence of a significant association between Talent Management and innovative work behaviour suggests that isolated, weakly structured or insufficiently institutionalised practices may be unable to produce meaningful organisational change. Innovation should therefore be regarded as a distal and context-dependent outcome rather than as a direct or inevitable consequence of Talent Management.

This result supports the proposed interpretative framework by indicating that outcomes may depend not only on the presence of individual practices but also on organisational maturity, strategic integration, enabling conditions and systematic evaluation. Organisations implementing similar practices may consequently achieve different outcomes depending on their leadership, culture, resources, workforce context and capacity to integrate Talent Management within organisational strategy.

Although Talent Management is frequently presented as contributing to quality of care, patient safety and health system sustainability, these relationships were not directly established within the evidence included in this review. Patient-related and clinical outcomes should therefore be considered theoretically plausible distal outcomes that require empirical testing rather than demonstrated consequences of Talent Management.

### 4.5. The Missing Evaluation Architecture

Measurement remains one of the least developed areas of Talent Management in nursing. The Nurses’ Perception of Talent Management Scale developed by Gül and Sönmez [33] was the only nursing-specific measurement instrument identified in this review. Its development represents an important advance in the assessment of nurses’ perceptions of organisational Talent Management practices.

The doctoral work developed by Gül [32] provided the conceptual and empirical basis for this instrument-development programme. The doctoral thesis by Gül [32] and the subsequent validation article by Gül and Sönmez [33] report the same instrument-development and validation study, conducted with the same sample of nurses across 12 hospitals in Istanbul, Turkey; the published article explicitly cites the doctoral thesis as its source. These two records were therefore treated as connected reports of a single underlying study, rather than as independent sources of evidence, when interpreting the contribution of this research programme to the review.

Although the Nurses’ Perception of Talent Management Scale provides a useful measure of nurses’ perceptions, a perception-based and unidimensional instrument cannot independently capture the full organisational architecture proposed in this review. Future evaluation approaches should distinguish between at least four different levels: the assessment of individual talent and potential; nurses’ perceptions of Talent Management practices; the implementation and maturity of organisational Talent Management programmes; and the resulting workforce, organisational and patient outcomes.

Importantly, no validated measurement instrument specifically designed for the early identification of individual nursing talent or high-potential nurses was identified in this review. The NPTMS should therefore not be interpreted as an individual talent-identification tool, as it assesses nurses’ perceptions of organisational Talent Management practices rather than individual talent or potential. This represents an important gap in the current evidence. Future research should determine which dimensions most appropriately capture individual nursing talent and potential and subsequently develop and psychometrically validate instruments capable of supporting Human Resources and nursing managers in its early and systematic identification.

These levels are conceptually distinct and should not be combined within a single construct. A comprehensive evaluation architecture should therefore integrate perception-based measures with objective indicators such as access to development opportunities, career progression, internal mobility, succession readiness, leadership pipeline strength, work engagement, turnover intention, actual retention and organisational performance. Where theoretically and methodologically appropriate, nursing-sensitive and patient outcomes should also be examined.

The limited availability of culturally adapted and multidimensional instruments currently restricts comparison across organisations and health systems. Future measurement research should therefore examine construct validity, cultural adaptation, measurement invariance and the capacity of instruments to discriminate between organisations at different levels of Talent Management maturity.

### 4.6. Progression Towards Organisational Talent Maturity

Building on the interpretative synthesis and the operational criteria defined in Section 2, this review proposes a Nursing Talent Management Maturity Model comprising five levels: Administrative Human Resource Management, Talent Practices, Integrated Talent Management, Strategic Talent Capability and Learning Health Organisation. The study-level assignments derived from these criteria are presented in Appendix C. The model introduces an organisational development perspective that is largely absent from the current nursing literature.

At the first level, workforce management is predominantly administrative, reactive and focused on recruitment, staffing, policy compliance and short-term operational needs. At the second level, explicit Talent Management practices begin to emerge in areas such as professional development, performance management and retention, but remain isolated, fragmented or limited to specific individuals, departments or programmes.

The third level is characterised by the coordination and integration of multiple Talent Management processes within a shared organisational structure. These may include talent identification, development, career pathways, performance evaluation, succession planning, leadership development and workforce planning, with increasing alignment across organisational functions.

At the fourth level, Talent Management becomes a strategic organisational capability, explicitly integrated with organisational strategy and workforce planning. Workforce planning, talent segmentation, leadership pipelines, engagement, retention and analytics are incorporated into organisational decision-making, and Talent Management is systematically evaluated through defined indicators.

At the fifth level, Strategic Talent Capability is further embedded within a Learning Health Organisation characterised by continuous organisational learning, data-informed feedback and systematic adaptation of Talent Management practices. At this level, workforce and organisational outcomes are continuously monitored and used to inform organisational learning and the ongoing development of nursing practice. No included study met all the operational criteria required for assignment to Level 5.

The maturity model provides a possible explanation for the heterogeneous findings identified across the included studies. Similar practices may be associated with different outcomes depending on whether they are implemented as isolated initiatives or as part of a more integrated and strategically aligned organisational capability. The model also suggests that the accumulation of individual practices does not necessarily indicate organisational maturity; increasing integration, strategic alignment, evaluation capacity and organisational learning distinguish the proposed levels.

The proposed model should, however, be interpreted as an evidence-informed conceptual proposition rather than as an empirically validated classification system. Its levels, indicators and assumptions regarding organisational progression require testing across different organisational, professional and cultural contexts. Organisations may also demonstrate characteristics of more than one level simultaneously, rather than progressing through a strictly linear sequence.

### 4.7. Towards an Inclusive and Differentiated Talent Philosophy

The findings also raise an important question regarding the philosophy underpinning Talent Management in nursing. A strictly exclusive approach, focused on a restricted group of high-performing or high-potential professionals, may conflict with professional values of equity, collective competence and access to development. It may also privilege visible leadership potential while overlooking less visible forms of talent, including advanced clinical expertise, education, research, innovation and digital capability.

Conversely, a fully undifferentiated approach may limit the organisation’s capacity to direct resources towards strategically critical roles, specialised areas or succession priorities. The evidence therefore appears more compatible with an inclusive but differentiated philosophy. Under this approach, all nurses should have access to meaningful professional development, while additional or specialised investment may be directed towards specific career aspirations, critical organisational roles, leadership responsibilities and areas of advanced expertise.

Such differentiation should be based on transparent, equitable and revisable criteria. This is particularly important because the included studies provided limited information regarding how talent is identified, which actors participate in identification decisions, what criteria are used and how potential bias is prevented. Without clear criteria, Talent Management may reproduce managerial subjectivity, create perceptions of favouritism and undermine professional trust.

Future nursing Talent Management models should therefore recognise multiple forms of talent and support differentiated career pathways in clinical practice, leadership, education, research, innovation, advanced practice and digitally enabled nursing. This would allow Talent Management to reflect the diversity of nursing contribution rather than equating talent primarily with progression into formal management roles.

### 4.8. Theoretical Contribution

This review advances the conceptual understanding of Talent Management in hospital nursing in four principal ways. First, it distinguishes organisational antecedents, core Talent Management processes, relational and motivational mechanisms, and reported outcomes, thereby reducing the conceptual overlap observed in the existing literature.

Second, it positions Talent Management as a dynamic organisational capability rather than as a collection of isolated human resource practices. This interpretation explains why the presence of individual development or retention interventions may not be sufficient to generate sustainable workforce outcomes.

Third, the review proposes a maturity model describing how organisations may progress from administrative workforce management towards an integrated strategic talent capability embedded within a learning health organisation. This organisational development perspective helps explain why similar practices may produce different results across institutional contexts.

Fourth, the proposed interpretative framework identifies a set of relationships that can be empirically tested in future research. These include the potential role of leadership, culture and career structures as organisational antecedents; engagement, psychological contract fulfilment, social capital and professional confidence as intermediate mechanisms; and retention, leadership development, innovation and workforce sustainability as potential outcomes.

Collectively, these contributions extend the current literature beyond descriptive consolidation by offering an integrated conceptual architecture and a testable organisational maturity proposition for Talent Management in hospital nursing.

### 4.9. Implications for Practice

The findings of this review have implications for nursing leadership, hospital management and human resource governance. No Portuguese national Talent Management model for hospital nurses was identified through the search strategy used in this review, highlighting an opportunity to examine the applicability of the proposed framework within the Portuguese health system and other Portuguese-speaking contexts.

Hospital and nursing leaders should begin by defining an explicit organisational philosophy of talent and clarifying whether development opportunities are intended for all nurses, selected groups or a differentiated combination of both. Critical nursing capabilities and strategically relevant roles should then be identified using transparent and equitable criteria.

Talent identification, development, deployment, recognition, succession planning and retention should be integrated rather than managed as unrelated activities. Hospitals should create clear clinical and leadership career pathways, structured mentoring opportunities, academic–practice partnerships and mechanisms for professional participation and recognition. Talent Management should also recognise expertise in clinical practice, education, research, innovation and digital transformation, rather than focusing exclusively on management succession.

Implementation should be accompanied by an evaluation system combining nurses’ perceptions with objective indicators. Relevant measures may include participation in development programmes, internal mobility, career progression, succession readiness, engagement, intention to leave, actual turnover and retention. Evidence concerning care quality and patient safety remains insufficient; these outcomes should therefore be evaluated rather than assumed.

Health policies, regulatory arrangements and resource availability may shape the implementation and sustainability of Talent Management practices. Organisational models should consequently be adapted to the characteristics of each health system while maintaining principles of transparency, inclusion, fairness and accountability. These dimensions were insufficiently addressed in the identified literature and should be incorporated explicitly into future implementation models.

### 4.10. Implications for Future Research

The proposed interpretative framework and maturity model provide several directions for future research. Conceptual studies should continue to clarify the meaning of talent in nursing and examine whether talent should be defined through competence, potential, contribution, professional aspiration or a combination of these dimensions.

Measurement research should prioritise the development and validation of multidimensional instruments capable of assessing organisational Talent Management in nursing. Such instruments should distinguish nurses’ perceptions from organisational implementation, maturity and outcomes, and should be culturally adapted and tested across different health systems.

The proposed maturity model requires empirical testing. Future studies should define observable indicators for each maturity level and determine whether organisations progress sequentially through the proposed stages or may exhibit characteristics of several levels simultaneously. Comparative case studies could initially examine how organisations at different maturity levels implement and evaluate Talent Management.

Longitudinal, multicentre and mixed-methods studies are required to test the relationships between organisational antecedents, Talent Management processes, relational mechanisms and outcomes. Studies should assess whether changes in leadership, development structures and recognition practices are followed by changes in engagement, psychological contract fulfilment, organisational commitment and retention.

Future research should distinguish retention intention from actual retention and incorporate objective workforce indicators, including turnover, internal mobility, career progression, succession readiness, absenteeism and workforce stability. Multilevel studies should also examine whether organisational Talent Management capability is associated with nursing-sensitive and patient outcomes, as this relationship remains theoretically plausible but empirically underexplored.

The mediating and moderating roles of organisational culture, leadership climate, professional career stage, generational differences and national health system characteristics should also be examined. Particular attention should be given to equity in talent identification and access to development opportunities, including the risk that exclusive approaches may reinforce existing professional inequalities.

Future updates of this review, or complementary reviews, should consider extending the search to multidisciplinary and management-oriented databases, such as Scopus, Web of Science, Embase and relevant business-management databases, in order to capture additional conceptual and empirical contributions from the organisational behaviour and human resource management literature that may not be indexed in nursing-specific or biomedical databases.

### 4.11. Strengths and Limitations

The main contribution of this scoping review is the integration of a fragmented body of evidence into an interpretative structure and an evidence-informed maturity model for Talent Management in hospital nursing. The review was conducted using recognised methodological and reporting guidance and included evidence from different methodological, organisational and geographical contexts.

However, several limitations should be considered. First, the evidence base was small and conceptually heterogeneous, including empirical studies, conceptual analyses, reviews, methodological studies, a practice article and a doctoral thesis. These sources differ in their evidential weight and may include conceptual or empirical overlap.

Second, no formal methodological quality appraisal was conducted, consistent with the chosen scoping review methodology. Consequently, the relative robustness of the included findings could not be incorporated formally into the synthesis.

Third, most empirical studies used cross-sectional, qualitative or perception-based designs. This limits causal inference regarding the relationships between Talent Management practices, relational mechanisms and workforce outcomes. Much of the available evidence also concerns intention to remain rather than objectively measured retention or turnover.

Fourth, the concentration of studies in a limited number of countries and health systems restricts the transferability of the findings and proposed maturity levels. Cultural, professional, regulatory and organisational differences may influence how talent is defined, identified and managed.

Fifth, evidence concerning innovation, organisational performance, care quality and patient outcomes was limited or inconsistent. These outcomes should therefore not be interpreted as demonstrated effects of Talent Management.

Sixth, the search was concentrated in nursing, health sciences and evidence-based medicine databases (MEDLINE, CINAHL, Nursing & Allied Health Collection, CENTRAL, CDSR, MedicLatina), complemented by grey literature sources, and did not include multidisciplinary or management-oriented databases such as Scopus, Web of Science, Embase or relevant business-management databases. This decision reflected the review’s specific focus, as defined a priori in the registered protocol, on Talent Management as applied to nursing practice within hospital settings. Nevertheless, it may have limited the retrieval of relevant conceptual or empirical contributions originating from the organisational behaviour and human resource management literature, representing a risk of incomplete evidence retrieval that should be considered when interpreting the findings of this review.

The possibility of publication bias should also be acknowledged, as studies reporting positive associations may be more likely to be published than studies reporting neutral or negative findings. Language restrictions and the dynamic development of the field may have further limited the comprehensiveness of the evidence identified.

Finally, the interpretative framework and maturity model were derived from a small and heterogeneous evidence base and have not yet been empirically validated. They should therefore be understood as evidence-informed conceptual propositions intended to support future model refinement, empirical testing and context-sensitive implementation.

Overall, the evidence suggests that Talent Management in hospital nursing is more appropriately understood as a progressive organisational capability than as a collection of isolated human resource practices. The interpretative structure and maturity model proposed in this review extend current understanding by explaining how organisational antecedents, Talent Management processes and relational mechanisms may interact to influence workforce outcomes. The most consistently reported associations concerned engagement, organisational attachment and retention intention, whereas actual retention, innovation, organisational performance and patient outcomes were less frequently examined or yielded inconsistent findings. The proposed models therefore provide a foundation for future empirical validation rather than definitive evidence of effectiveness.

## 5. Conclusions

This scoping review mapped and interpretatively synthesised the available scientific evidence on Talent Management in hospital nursing. Beyond identifying the principal conceptual dimensions, organisational strategies and measurement approaches described in the literature, the review integrated the available evidence into an interpretative framework and proposed a Nursing Talent Management Maturity Model, thereby extending the conceptual understanding of Talent Management in nursing beyond descriptive evidence mapping.

The interpretative synthesis suggests a possible progression from predominantly fragmented and reactive Talent Management practices towards more integrated and strategically aligned organisational approaches. This proposed progression is characterised by the interaction of organisational antecedents, Talent Management processes, relational and motivational mechanisms, and workforce outcomes. The proposed maturity model reflects this progression by conceptualising organisational development from administrative human resource management to a learning health organisation in which Talent Management becomes embedded within organisational strategy and continuous workforce development.

The review also highlights persistent conceptual and methodological heterogeneity across the literature. Variability in definitions of talent, Talent Management philosophies, organisational models and measurement approaches continues to limit comparison between studies and the cumulative development of knowledge. The interpretative framework proposed in this review represents an initial step towards greater conceptual coherence by distinguishing organisational antecedents, Talent Management processes, relational and motivational mechanisms, and workforce outcomes.

The most consistently reported associations concerned work engagement, organisational commitment and retention intention, whereas actual retention, innovation, organisational performance and patient outcomes were less frequently examined or yielded inconsistent findings. The proposed framework and maturity model should therefore be regarded as evidence-informed conceptual propositions requiring empirical validation through longitudinal, implementation and multilevel research.

Overall, this review contributes to the advancement of Talent Management in hospital nursing by providing an integrated conceptual architecture that may guide future research, organisational implementation and policy development. Rather than viewing Talent Management as a collection of isolated human resource practices, the review positions it as a dynamic organisational capability capable of supporting workforce sustainability, professional development and strategic organisational learning across increasingly complex healthcare systems.

## Figures and Tables

**Figure 1:**
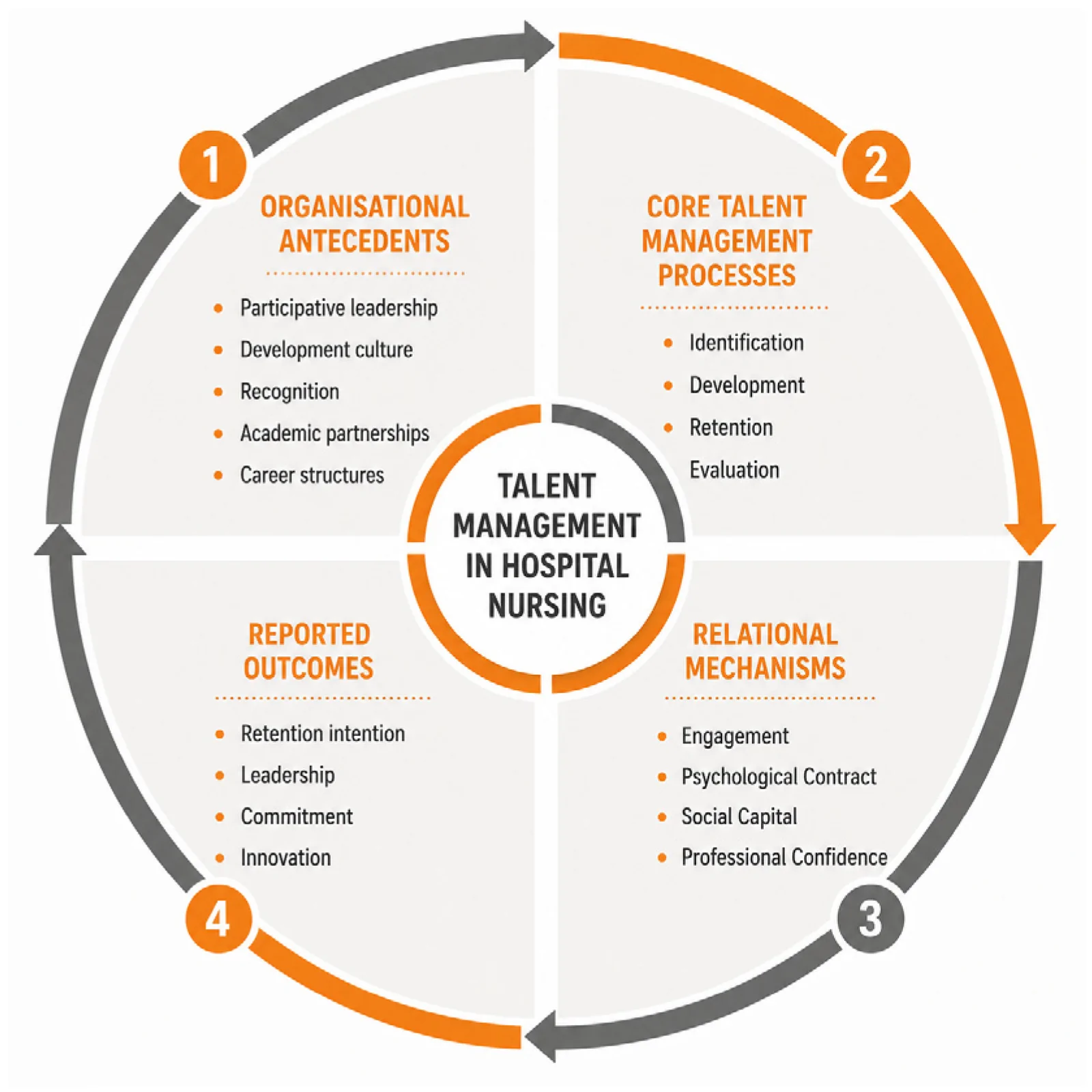
Interpretative structure of Talent Management in hospital nursing derived from the evidence synthesis.

**Figure 2:**
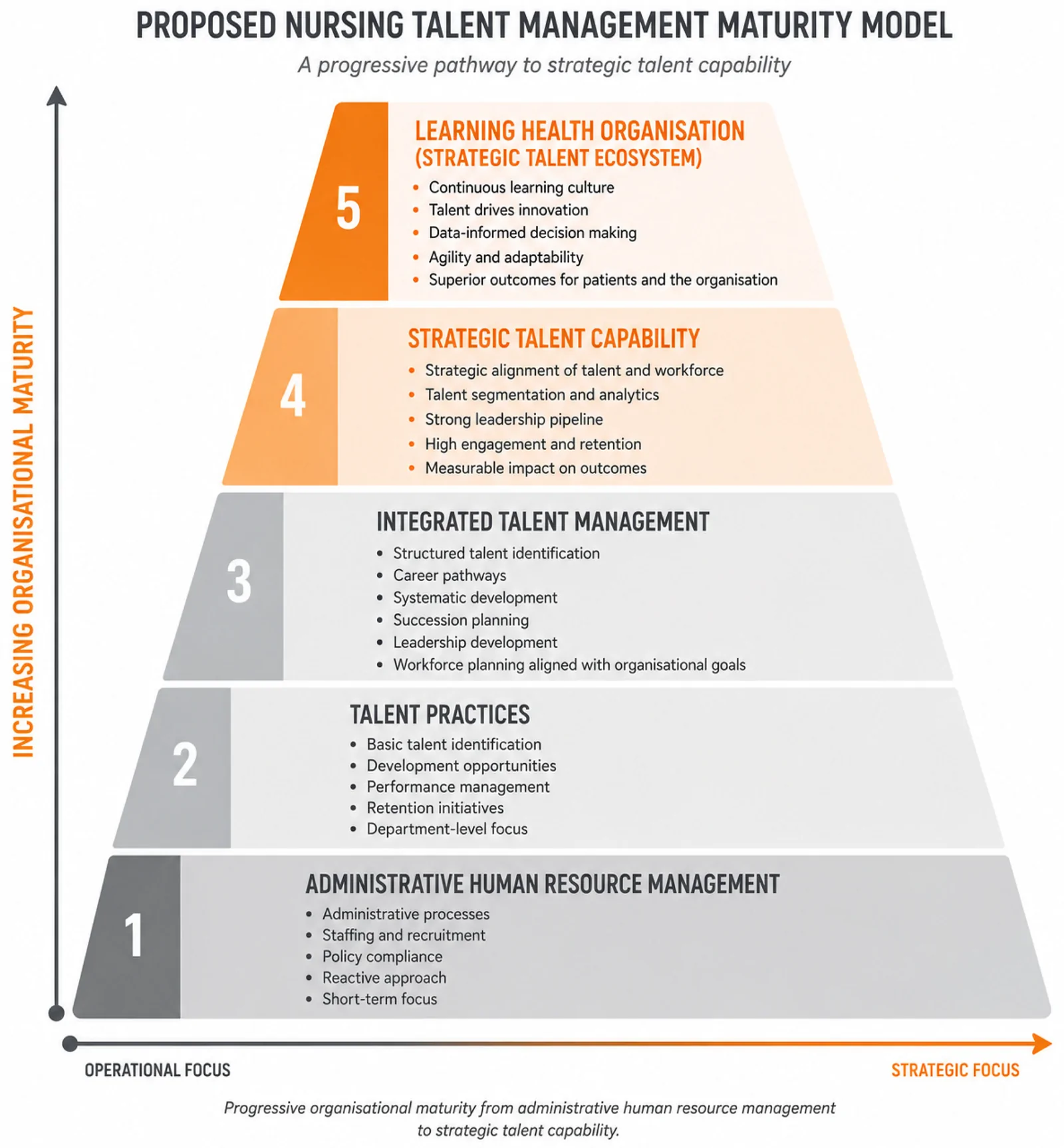
Proposed Nursing Talent Management Maturity Model derived from the interpretative synthesis of the included studies.

**Table 1:** Eligibility criteria organised according to the PCC framework.

PCC Component	Inclusion Criteria	Exclusion Criteria
Population	Nurses (regardless of professional category or level of differentiation) working in the hospital setting (public, private or university hospitals)	Nursing students; non-nursing health professionals when they constitute the main study population
Concept	Studies addressing organisational approaches to Talent Management, including conceptual models, implementation strategies, organisational practices, and processes of talent identification, development, retention and evaluation.	Studies focused exclusively on clinical or technical competencies without an explicit Talent Management framework.
Context	Hospital organisations (public or private); inpatient setting	Studies conducted exclusively in primary care, community or non-clinical settings
Study type	Empirical studies (quantitative, qualitative or mixed methods); literature reviews (systematic, scoping, integrative); case studies; dissertations and theses; grey literature with full text available;	Opinion articles; editorials; letters to the editor; documents without full-text access; studies published in other languages; duplicates

**Table 2:** Search strategies by database and results.

Database	*n*	Search Strategy
MEDLINE (PubMed)	6927	(Nurses[Title/Abstract] OR Nurses[MeSH Terms] OR Nurse[MeSH Terms] OR Nurse[Title/Abstract] OR “Personnel, Nursing”[Title/Abstract] OR “Personnel, Nursing”[MeSH Terms] OR “Nursing Personnel”[MeSH Terms] OR “Nursing Personnel”[Title/Abstract] OR “Registered Nurses”[Title/Abstract] OR “Registered Nurses”[MeSH Terms] OR “Registered Nurse”[MeSH Terms] OR “Registered Nurse”[Title/Abstract]) AND (“Models, Organizational”[Title/Abstract] OR “Models, Organizational”[MeSH Terms] OR “Organizational Models”[MeSH Terms] OR “Organizational Models”[Title/Abstract] OR “Organizational Model”[Title/Abstract] OR “Organizational Model”[MeSH Terms] OR Talent[MeSH Terms] OR Talent[Title/Abstract] OR Talents[Title/Abstract] OR Talents[MeSH Terms]) AND (“Personnel Administration, Hospital”[MeSH Terms] OR “Personnel Administration, Hospital”[Title/Abstract] OR “Hospital Personnel Administration”[Title/Abstract] OR “Hospital Personnel Administration”[MeSH Terms] OR “Personnel Management, Hospital”[MeSH Terms] OR “Personnel Management, Hospital”[Title/Abstract] OR “Management, Hospital Personnel”[Title/Abstract] OR “Management, Hospital Personnel”[MeSH Terms] OR “Administration, Hospital Personnel”[MeSH Terms] OR “Administration, Hospital Personnel”[Title/Abstract] OR “Personnel, Hospital, Organization and Administration”[Title/Abstract] OR “Personnel, Hospital, Organization and Administration”[MeSH Terms] OR Administration[MeSH Terms] OR Administration[Title/Abstract] OR “Hospital Personnel Organization and Administration”[Title/Abstract] OR “Hospital Personnel Organization and Administration”[MeSH Terms])
CINAHL^®^ Plus with Full Text (EBSCO)	335	(TI hospital OR AB hospital OR MH hospital) AND (MH modelos organizacionales OR TI modelos organizacionales OR AB modelos organizacionales OR MH personnel management OR TI personnel management OR AB personnel management) AND ((MM talent OR TI talent OR AB talent OR AB talent management OR MH Talent management OR TI Talent management) OR (MH modelos organizacionales OR TI modelos organizacionales OR AB modelos organizacionales OR MH personnel management OR TI personnel management OR AB personnel management)) AND (TI nurs* OR AB nurs* OR MH nurs*)
Nursing & Allied Health Collection (EBSCO)	1132	TI hospital OR AB hospital OR MH hospital) AND (MH modelos organizacionales OR TI modelos organizacionales OR AB modelos organizacionales OR MH personnel management OR TI personnel management OR AB personnel management) AND ((MM talent OR TI talent OR AB talent OR AB talent management OR MH Talent management OR TI Talent management) OR (MH modelos organizacionales OR TI modelos organizacionales OR AB modelos organizacionales OR MH personnel management OR TI personnel management OR AB personnel management)) AND (TI nurs* OR AB nurs* OR MH nurs*)
Cochrane Central Register of Controlled Trials (CENTRAL)	1	TI hospital OR AB hospital OR MH hospital) AND (MH modelos organizacionales OR TI modelos organizacionales OR AB modelos organizacionales OR MH personnel management OR TI personnel management OR AB personnel management) AND ((MM talent OR TI talent OR AB talent OR AB talent management OR MH Talent management OR TI Talent management) OR (MH modelos organizacionales OR TI modelos organizacionales OR AB modelos organizacionales OR MH personnel management OR TI personnel management OR AB personnel management)) AND (TI nurs* OR AB nurs* OR MH nurs*)
Cochrane Database of Systematic Reviews (CDSR)	0	TI hospital OR AB hospital OR MH hospital) AND (MH modelos organizacionales OR TI modelos organizacionales OR AB modelos organizacionales OR MH personnel management OR TI personnel management OR AB personnel management) AND ((MM talent OR TI talent OR AB talent OR AB talent management OR MH Talent management OR TI Talent management) OR (MH modelos organizacionales OR TI modelos organizacionales OR AB modelos organizacionales OR MH personnel management OR TI personnel management OR AB personnel management)) AND (TI nurs* OR AB nurs* OR MH nurs*)
MedicLatina (EBSCO)	13	TI hospital OR AB hospital OR MH hospital) AND (MH modelos organizacionales OR TI modelos organizacionales OR AB modelos organizacionales OR MH personnel management OR TI personnel management OR AB personnel management) AND ((MM talent OR TI talent OR AB talent OR AB talent management OR MH Talent management OR TI Talent management) OR (MH modelos organizacionales OR TI modelos organizacionales OR AB modelos organizacionales OR MH personnel management OR TI personnel management OR AB personnel management)) AND (TI nurs* OR AB nurs* OR MH nurs*)
RCAAP (grey literature)	108	“modelo de gestão de talentos” OR “gestão de talentos” AND enfermagem
LILACS (via BVS)	18	modelo de gestão de talento [words] AND enfermagem [words]
SciELO	0	(ab:(talento)) AND (ti:(talento)) AND (enfermagem)
DANS Easy (grey literature)	0	talent management model [title words] and talent management model [abstract words] and talent management model [Subject Descriptor]

**Table 3:** Talent management components identified in the narrative analysis.

Component	Studies Addressing the Component (Ref.)	No. of Independent Studies *
Talent identification	[33,34]	2
Talent development	[33,35,36,40,41]	5
Talent retention	[35,36,37,38,39,40,41]	7
Talent evaluation	[32,33]	1

* Counts represent independent underlying studies rather than eligible reports. References [33,40] are connected reports arising from the same sample and research programme and were therefore counted once.

**Table 4:** Main outcomes reported in the included literature.

Organisational Outcome	Studies Reporting the Outcome
Nurse retention	[36,37,38,39,40]
Work engagement	[39,40]
Leadership development	[34,35]
Strengthening of the psychological contract	[37]
Innovative behaviour	[42]

## Data Availability

No new data were created in this study. Data sharing is not applicable, as this article is a scoping review synthesising data from previously published studies, all of which are cited within the article.

## Supplementary Materials

The following supporting information can be downloaded at: https://www.mdpi.com/article/10.3390/nursrep16100346/s1, Table S1: Preferred Reporting Items for Systematic reviews and Meta-Analyses extension for Scoping Reviews (PRISMA-ScR) Checklist.

## Public Involvement Statement

No public involvement in any aspect of this research.

## Guidelines and Standards Statement

This study was conducted and reported in accordance with the Preferred Reporting Items for Systematic Reviews and Meta-Analyses extension for Scoping Reviews (PRISMA-ScR) guidelines and the Joanna Briggs Institute (JBI) methodology for scoping reviews.

## Appendix B

**Table A1:** Characteristics of included records (*n* = 11).

Title	Authors/Year/Country	Study Type	Main Objective	Main Results
The development of nurses’ perception of talent management scale and a model testing for perception of talent management	Gül [32] 2024 Türkiye	Methodological study	To develop and test a perception model of Talent Management in nursing, forming the empirical basis of the NPTMS scale.	Direct conceptual and empirical basis of the NPTMS. Represents the only model tested specifically for the perception of Talent Management among nurses
Nurses’ perception of talent management scale (NPTMS): development, validation and psychometric properties	Gül and Sönmez [33] 2025 Türkiye	Methodological	To develop and validate a scale to measure nurses’ perceptions of talent management in the nursing profession.	Unidimensional 26-item scale (NPTMS) with excellent validity and reliability indicators, suitable for assessing perceptions of talent management practices.
Strategy to stay ahead of the curve: A concept analysis of talent management	Nowak and Scanlan [34] 2020 Canada	Concept analysis	To clarify the concept of talent management as an approach to identifying, strengthening and supporting nursing leaders.	Lack of conceptual clarity in the literature. Critical attributes: identification of emerging leaders and development of leadership competencies for organisational stability.
Applying talent management to nursing	Haines [35] 2013 United Kingdom	Practice article	To identify talent development priorities in nursing within the NHS, based on consultation with nurses and nursing students.	Highlights critical factors of Talent Management in nursing: team leadership, effective performance evaluation, clear career pathways, nurse involvement and recognition
Academic practice partnership: Transitions into practice for new nurses	Cadmus et al. [36] 2014 USA	Qualitative	To describe and evaluate a transition programme for new nurses through an academic–hospital partnership, focusing on preceptorship, satisfaction and retention.	Academic–hospital partnerships strengthen professional transition, reduce early turnover and increase the confidence and competence of newly qualified nurses.
Talent management in VUCA health environment: nurses’ psychological contract, cordial relation and generation	Ellethiey et al. [37] 2024 Egypt	Quantitative	To analyse how talent management practices in a VUCA environment influence nurses’ fulfilment of the psychological contract, cordial relations and the moderating effect of generation.	Talent Management perceptions were associated with psychological contract fulfilment; cordial relations were reported as a mediating factor, with generational moderation. Generation Z showed a counterproductive moderating effect compared with Baby Boomers.
A qualitative study exploring the influence of a talent management initiative on registered nurses’ retention intentions	Fisher et al. [38] 2022 United Kingdom	Qualitative	To investigate the influence of a Talent Management initiative within an English NHS Trust on the retention intentions of registered nurses (*n* = 8 interviews).	Participants perceived the Talent Management initiative as supporting their retention intentions; networks and networking emerged as relevant forms of social capital.
Nurses’ perception toward talent management and its relationship to work engagement and retention	Elhaddad et al. [39] 2020 Egypt	Quantitative	To analyse nurses’ perceptions of Talent Management and its relationship with work engagement and retention.	Shows that a positive perception of Talent Management practices among nurses is associated with greater engagement and retention intention
Recruitment and Retention Strategies: A Magnet Hospital Prevention Model	Upenieks [40] 2005 USA	Review	To present a preventive model to support managers in developing recruitment and retention strategies in hospitals, based on the Magnet model.	Magnet factors (participative leadership, autonomy, recognition, development) associated with greater attraction and retention. Recommendations include partnerships with nursing schools and flexible working.
Talent Management in Healthcare: A Systematic Qualitative Review	Mitosis et al. [41] 2021 Cyprus	Systematic review	To identify the factors related to talent management practices in nursing.	Very limited number of studies on Talent Management in healthcare. Talent Management strategies are pivotal for the sustainable development of organisations, benefiting nurses, patients and teams.
Talent Management and its Impact on Innovative Work Behaviour among Registered Nurses in Public Hospitals of Malawi	Dzimbiri and Molefi [42] 2021 Malawi	Quantitative	To analyse the impact of talent management on innovative behaviour among nurses in public hospitals in Malawi (*n* = 312).	Talent Management practices had no significant impact on innovation, partly due to the absence of clear and structured policies within the organisations.

Note: Gül [32] and Gül and Sönmez [33] report connected stages of the same underlying research programme (doctoral thesis and its subsequent validation publication, respectively), conducted with the same sample.

**Table A2:** Other characteristics of included studies.

Ref.	Sample/Participants	Setting	Recruitment	Measures/Data Collection	Analysis	Detailed Numerical/Qualitative Findings
[32]	Nurses from 12 hospitals in Istanbul, Türkiye; same underlying sample as the later validation study [40] (convenience sampling).	12 hospitals, Istanbul, Türkiye.	Convenience sampling.	Early item pool for a nurses’ perception-of-Talent-Management instrument, later refined and published as the NPTMS [40].	Exploratory scale-development procedures and model testing.	Provided the conceptual and empirical basis for the NPTMS; methodologically and sample-wise continuous with [40] (see note below and Section 4.5—same research programme, not an independent source of evidence).
[33]	*n* = 918 nurses (nEFA = 422; nCFA = 496); convenience sampling.	12 hospitals, Istanbul, Türkiye.	Convenience sampling; data collected September–April 2022.	Newly developed Nurses’ Perception of Talent Management Scale (NPTMS, 26 items).	Scale-development and psychometric validation (EFA, CFA, reliability testing).	Unidimensional 26-item scale with adequate/strong validity and reliability indicators; same underlying sample and research programme as [33] (see note below and Section 4.5).
[34]	N/A—concept analysis (no primary human sample); literature corpus retrieved from CINAHL, MEDLINE, PubMed, Business Source Premier, Canadian Major Dailies and Management & Organization Studies.	N/A (literature-based).	N/A.	N/A (no data-collection instrument).	Walker and Avant concept-analysis method (defining attributes, antecedents, consequences, empirical referents).	Lack of conceptual clarity in the literature; critical attributes identified as identification of emerging leaders and development of leadership competencies for organisational stability.
[35]	Nurses consulted informally; sample size not reported in the published practice article.	NHS, England (specific Trust not identified in the source).	Informal consultation; no structured sampling procedure reported.	N/A—non-empirical practice commentary; no named instrument.	Narrative/expert commentary; no formal qualitative or quantitative analysis reported.	Identifies critical factors for Talent Management in nursing: team leadership, performance evaluation, clear career pathways, nurse involvement and recognition.
[36]	Newly qualified nurses participating in an academic–hospital transition-to-practice programme	Robert Wood Johnson University Hospital, New Brunswick, NJ, USA, in partnership with Rutgers University College of Nursing.	Programme participants (residency cohort); not a research sampling procedure.	Programme-evaluation indicators (satisfaction, retention, transition experience); no named validated instrument reported.	Descriptive programme evaluation/narrative report.	Academic–hospital partnership strengthens professional transition, reduces early turnover and increases confidence and competence of newly qualified nurses.
[37]	*n* = 375 nurses (of 600 total nurses); non-probability convenience sampling.	Three private hospitals, Alexandria, Egypt.	Non-probability convenience sampling; cross-sectional design.	Structured questionnaire assessing Talent Management, psychological contract fulfilment, cordial relations and generational cohort.	Cross-sectional moderation–mediation modelling.	Low mean psychological contract fulfilment (8.89 ± 3.93) and moderate cordial relation (185.11 ± 27.02); Talent Management positively predicted cordial relations and psychological contract fulfilment; cordial relations partially mediated this relationship; generation moderated the association (Baby Boomers > Generation Z).
[38]	*n* = 8 registered nurses (semi-structured interviews).	English acute NHS Trust.	Purposive sampling of nurses who had participated in a Talent Management initiative.	Semi-structured interview guide; data collected July 2019.	Qualitative thematic analysis.	The Talent Management initiative positively influenced retention intentions; networks and networking functioned as social capital facilitating nurse retention.
[39]	*n* = 400 nurses; convenience sampling.	Menoufia University Hospital, Shebin El-Kom, Egypt.	Convenience sampling; descriptive-correlational design.	Structured questionnaire on perception of Talent Management, work engagement and retention.	Descriptive-correlational statistics.	Low perception of Talent Management; more than half of nurses reported low work engagement; majority reported low retention; positive, highly significant correlations between perception of Talent Management, engagement and retention.
[40]	N/A—narrative, literature-based review/model description; no primary empirical sample.	N/A (draws on Magnet-recognised hospitals generally; not a single site).	N/A.	N/A (no primary data-collection instrument).	Narrative synthesis of the Magnet-model literature.	Magnet factors (participative leadership, autonomy, recognition, development) associated with greater attraction and retention; recommends partnerships with nursing schools and flexible working.
[41]	24 included studies (of 684 identified); pooled participant sample *n* = 5159 (secondary systematic review, not a single primary sample).	Multi-country healthcare organisations; primary studies published 2010–2020.	N/A (secondary systematic review of primary studies).	PRISMA-guided systematic search of PubMed, CINAHL, CDSR and Health Source–Nursing Academic Edition; quality appraisal of included studies.	Qualitative content analysis.	Very limited body of primary research on Talent Management in healthcare; factors grouped into nine categories (e.g., Attraction, Development, Preservation, Performance Assessment, Succession Planning, Leadership).
[42]	*n* = 312 registered nurses.	Public hospitals, Malawi.	Not Reported	Structured questionnaire on Talent Management practices and innovative work behaviour.	Quantitative regression/correlation analysis.	No significant impact of Talent Management practices on innovative work behaviour, attributed to the absence of structured organisational Talent Management policies.

## Appendix C

**Table A3:** Mapping between included studies, framework components and proposed maturity levels.

Study (Ref.)	Core TM Process (es)	Framework Component	Proposed Maturity Level	Rationale
Gül [32]	Evaluation	Measurement instrument	Not assigned	Measurement-focused report underpinning the development of a Talent Management perception instrument; insufficient evidence of multiple Talent Management processes coordinated within a shared organisational structure or programme to support assignment to a maturity level
Gül and Sönmez [33]	Identification, Development, Evaluation	Measurement instrument	Not assigned	Measurement-focused report validating a unidimensional perception-based Talent Management instrument (NPTMS); insufficient evidence of coordinated multiple Talent Management processes within an organisational structure or programme to support assignment to a maturity level
Nowak and Scanlan [34]	Identification	Core process/conceptual antecedent	Level 2—Talent Practices	Concept analysis focused on identifying and developing emerging leaders; individual, not yet organisation-wide.
Haines [35]	Development, Retention	Core process	Level 2—Talent Practices	Identifies fragmented practices (leadership, evaluation, career pathways) not yet integrated.
Cadmus et al. [36]	Development, Retention	Antecedent (academic–practice partnership)	Level 3—Integrated Talent Management	Cross-institutional coordination (academic–hospital partnership).
Ellethiey et al. [37]	Retention	Relational/motivational mechanism	Not assigned	Relational and motivational mechanism study examining associations between Talent Management perceptions, psychological contract fulfilment, interpersonal relations and generational differences; insufficient evidence of explicit integration with organisational strategy, workforce planning and systematic evaluation to support assignment to Level 4
Fisher et al. [38]	Retention	Core process + mechanism (social capital)	Level 3—Integrated Talent Management	Trust-level Talent Management initiative coordinating retention efforts.
Elhaddad et al. [39]	Retention	Mechanism (engagement) + outcome	Not assigned	Association study linking nurses’ perceptions of Talent Management practices with work engagement and retention intention; insufficient evidence of multiple Talent Management processes coordinated within a shared organisational structure or programme to support assignment to Level 3.
Upenieks [40]	Development, Retention	Antecedent + outcome	Level 3—Integrated Talent Management	The Magnet model integrates multiple Talent Management-related practices, including leadership, recognition, professional development and workforce planning, within a coordinated organisational approach; however, the available evidence does not explicitly demonstrate the systematic evaluation required for assignment to Level 4.
Mitosis et al. [41]	Development, Retention	Cross-cutting / conceptual synthesis	Foundational (cross-level)	Systematic review synthesising practices across contexts; not attributable to a single level.
Dzimbiri and Molefi [42]	(outcome: innovative behaviour)	Outcome (absence of effect)	Level 1—Administrative HR Management	Explicitly attributes null effect to the absence of structured Talent Management policies.

Proposed maturity levels reflect the review team’s interpretative synthesis and should be regarded as evidence-informed propositions rather than as an empirically validated classification system. Study-level assignments were made only where the included source provided sufficient organisational-level evidence to apply the operational criteria defined in Section 2 (Data Analysis and Presentation); sources focused primarily on measurement or associations without sufficient evidence of organisational integration were not assigned to a maturity level. These assignments are presented to enhance the transparency and traceability of the framework and maturity model reported in Section 3.6 and Section 4.6.

## Appendix D

**Table A4:** Derivation table linking extracted findings, codes, categories and higher-order constructs.

Study (Ref.)	Extracted Finding (Illustrative)	Code	Category	Higher-Order Construct
Gül [32]	Conceptual and empirical model of nurses’ perception of Talent Management	Perception-based evaluation model	Talent evaluation (a priori category)	Core process: Evaluation/measurement instrument
Gül and Sönmez [33]	Validated unidimensional 26-item scale (NPTMS) with adequate psychometric properties	Perception-based Talent Management measurement	Measurement instrument (a priori category: evaluation)	Core process: Evaluation/measurement architecture
Nowak and Scanlan [34]	Identification of emerging leaders and development of leadership competencies for organisational stability	Leadership identification and development	Talent identification (a priori category)	Core process: Identification
Haines [35]	Team leadership, performance evaluation, career pathways, participation and recognition as critical factors	Fragmented enabling practices	Organisational antecedents (inductive category)	Antecedent
Cadmus et al. [36]	Academic–hospital partnerships strengthen transition, reduce early turnover, increase confidence and competence	Academic–practice partnership	Organisational antecedent (inductive category)	Antecedent + core process (Development/Retention)
Ellethiey et al. [37]	Talent Management perceptions associated with psychological contract fulfilment; cordial relations reported as mediator; generational moderation.	Psychological contract mechanism	Relational/motivational mechanism (inductive category)	Mechanism
Fisher et al. [38]	Participants perceived the Talent Management initiative as supporting retention intentions, with networks and social capital identified as relevant relational factors.	Social capital mechanism	Relational mechanism (inductive category)	Mechanism + outcome (retention intention)
Elhaddad et al. [39]	Positive perception of Talent Management associated with greater engagement and retention intention	Engagement–retention link	Mechanism–outcome pathway (inductive category)	Mechanism (engagement) + outcome
Upenieks [40]	Magnet factors associated with greater attraction and retention; academic partnerships, flexible working	Magnet enabling architecture	Organisational antecedent (inductive category)	Antecedent + outcome
Mitosis et al. [41]	Very limited number of studies; Talent Management pivotal for sustainable organisational development	Field immaturity + strategic relevance	Conceptual gap (inductive category)	Cross-cutting/knowledge gap
Dzimbiri and Molefi [42]	No significant impact on innovative behaviour; attributed to absence of structured policies	Absence of institutionalisation	Outcome/negative case (inductive category)	Outcome (absence of effect) → informs maturity Level 1

A priori categories (talent identification, development, retention and evaluation) were adopted from pre-existing Talent Management models in the literature and used as the initial coding structure. All remaining categories, namely organisational antecedents, relational and motivational mechanisms, outcomes and the five levels of the proposed Maturity Model, emerged inductively from the iterative reading, comparison and synthesis of the included studies. This table presents one illustrative finding per study; it does not exhaust all codes generated during analysis and should be read together with Appendix C.
